# Editorial: Perceptions of Human-Animal Relationships and Their Impacts on Animal Ethics, Law and Research

**DOI:** 10.3389/fpsyg.2020.631238

**Published:** 2021-01-05

**Authors:** Marie Pelé, Jean-Yves Georges, Tetsuro Matsuzawa, Cédric Sueur

**Affiliations:** ^1^Anthropo-Lab, ETHICS EA7446, Lille Catholic University, Lille, France; ^2^Université de Strasbourg, CNRS, IPHC UMR 7178, Strasbourg, France; ^3^Kyoto University Institute for Advanced Study, Kyoto, Japan; ^4^Centre Européen d'Enseignement et de recherche en Éthique, Strasbourg, France; ^5^Institut Universitaire de France, Paris, France

**Keywords:** speciesism, animalism, animal conservation, animal consciousness, cognitive bias, one-health, animal law, animal ethics

Non-human animals live in ecosystems that are increasingly impacted by the growing human population, and have now developed relationships that mostly or partly depend on human societies. Although some of these relationships are positive and enable non-human animals to enjoy anthropized environments, most relationships with humans are negative and prove to be disastrous for non-human animals. Individuals are suffering and biodiversity is being lost at an unprecedented rate. However, human behavior varies, and people from non-industrialized societies behave differently from those living in WEIRD societies (Henrich et al., 2010). This includes their attitude toward animals, as shown in different approaches such as totemism or animism (Descola, 2019). Human perceptions of animal species in terms of their presence and function, and the potential co-use or sharing of their personal environment, depend on multiple sociocultural and biological factors. Humans usually make discriminations between animal species based on these perceptions. Donaldson and Kymlicka (2011) recently classified non-human animals into three categories according to their proximity with human beings, the role they fulfill and their distribution range, namely Wild, Domesticated and Liminal. Wild animals form their own communities and benefit from rights of sovereignty; domesticated animals are fully involved in human societies and may benefit from citizenship. Indeed, domesticated species have developed quite remarkable sociocognitive skills over the thousands of years they have coexisted with humans (Bhattacharjee et al.). Finally, liminal species are wild but live in the midst of human settlements and may benefit from resident status.

## Using Human Cognitive Biases in Animal Ethics

Discrimination between animal species is called speciesism, a term introduced in the 1970's by the psychologist Richard R Ryder (Sueur, 2019). Beside Kymlicka and Donaldson's categorization, a prime distinction was based on the phylogenetic proximity with human species (Miralles et al., 2019). In general, people show a greater preference for warm-blooded vertebrates than for invertebrates, but also show preferences for some species within a given taxon, for example preferring bees to wasps. Discrimination is also based on culture, as shown in the case of differences in diet and the traditional consumption of pork but not dog meat by Europeans, despite these two species being comparable at multiple levels such as body size, longevity or intelligence. Sueur et al. (2020) performed a survey to understand how humans displayed anthropomorphism toward animals, and showed that men and older participants are less likely to attribute human-like mental states to animals. Similarly, people who work with animals or have at least one pet at home demonstrated less anthropomorphism. Conversely, they found that members of animal protection associations attributed more intentions and mental states to animals than non-members. The emotions we feel for animals have important consequences for their welfare and for their conservation (Castillo-Huitrón et al.). While large predators and reptiles may trigger anger, fear, and disgust in some societies, they can produce emotions such as personal value-related happiness in people belonging to other ethnic groups. However, the excessive representation of some species on TV shows or in cartoons may create a biased perception of these animals, resulting in unintended detrimental effects on conservation efforts (Courchamp et al., 2018). Many humans express sadness about the threatening situations that animals are currently facing.

The way people perceive these animals, for instance in terms of how we coexist and interact with them, progresses as our societies, individual behavior and scientific knowledge evolve. For instance, the subject of slaughter sets the debate about defining an acceptable treatment of animals at an extremely low level, and the ethics around slaughtering have considerably evolved over at least the last half century in our western societies. Animal farming and meat consumption cause not only animal suffering but also global warming (Koneswaran and Nierenberg, 2008) and also play a role in the emergence and amplification of infectious diseases (Espinosa et al., 2020). Recently, there has been considerable investment in developing cell-based meat, an alternative meat production process that uses muscle cells cultivated in a bioreactor, thus eliminating the need to raise and slaughter animals. Heidemann et al. discuss the animal ethics impacts of cell-based and plant-based meat on human-animal interactions from animal welfare and rights perspectives, focusing on industrial meat production scenarios. Their hypothesis is that the insertion of cell-based meat in the global meat market may alleviate farm animal suffering and potentially restore resources for wild fauna by freeing up the land (one third of all fields) that is currently devoted to livestock. From a conservation perspective, empathy is subject to significant biases. This inflexible adherence to moral rules can result in a “do nothing” approach, as observed in the Australian case of biodiversity loss and the suffering of preys due to the proliferation of cats (although the perception of cats in Australia is now changing: see Riley, 2019; Woolley et al., 2020). Consequently, Griffin et al. consider that the Compassionate Conservation philosophy, which is based on empathy, should not be enshrined as a legalized guiding principle for conservation action as it could be detrimental to some species.

## Conceiving New Concepts in Animal Ethics

Current scientific research allows the development of animal ethics, animal legislation and animal research. However, some elements are still difficult to disentangle within the context of these new rules: How and why should/do we categorize animals, even if it would seem unrealistic to think in another way? Like Bentham (1907), Dzwonkowska suggests that we should not look at non-human animals from a human-related perspective, but from a suffering-related one (Nussbaum, 2004), thus calling for radical responsibility. Radical responsibility is a form of moral responsibility that extends our moral obligations to the point where we are responsible “for the unintended (and often unnoticed) consequences of our actions and our failures to act” (Dower, 1989, p. 18). Here, Dower introduces the idea that radical responsibility concerns not only our actions, but our indirect footprint through actions taken for us by others.

When it is strongly supported by citizens, moral consideration for animals is sometimes transformed into law. Should we give different animal species the same moral status and rights or should their rights differ according to their sentience? The crucial question is whether all species should be included in these animal rights categories, or whether they should be limited to vertebrates alone, or even only mammals. To answer these questions, we need to harmonize the different elements - biology, law, sociology, ethics and philosophy – involved in the moral consideration and protection of animals. Human and non-human factors contribute equally to how we consider animals. Castillo-Huitrón et al. propose that the management of culturally important animal species (particularly those regarded as frightening, dangerous, harmful and disgusting) should be included in national education programs and massive media campaigns. Kletty et al. discussed how to address the ethical limits and the societal perception of implemented conservation measures when dealing with the protection of an endangered species. Like culture, ethics change over time. An animal species can evolve from the status of a pest to one of a conservation flagship in three decades, but good conservation management requires societal demand and the involvement of citizens for the programs to succeed.

It is important to assess these conservation education programs, which can also be used to test the conservation education hypothesis suggesting that people are more likely to defend conservation if they have been exposed to knowledge about endangered species and ecosystems. If a positive result is observed, these programs need to be secured. Bowie et al. introduced novel methods to assess a small-scale program in the Democratic Republic of Congo and confirmed that conservation education has improved relevant knowledge and the attitudes people show about environmental and social issues and toward animals. Importantly, the authors discussed the important role children play in influencing their peers and family members to pursue pro-conservation behaviors.

The management of culturally important animal species closely follows the concept of Compassionate Conservation (Griffin et al.). The latter promotes “ethical” conservation practices, placing empathy and compassion and the moral principles of “first, do no harm” and “individuals matter” at the forefront of conservation practice. This means that environmental and animal ethics, which have often been opposed, must be combined (even if this can lead to dilemmas, as seen in the case of cats in Australia). The idea of combining environmental health with animal health has existed for about 10 years now and has a fundamental impact for human health, namely the so-called “One-health” concept (Destoumieux-Garzón et al., 2018). Human health, mental or physical, is impacted by the way we consider the environment and animals. When human health is endangered by the inappropriate use of biodiversity, it may result in a better protection of animals through measures such as wildlife trade and animal protection policies in China, which will likely be more strongly regulated in light of the recent SARS spread and Covid-19 pandemic (Bonilla-Aldana et al., 2020; Hemida and Ba Abduallah, 2020). Human fitness is measured in terms of health, and is only limited by the environment and human-animal interactions. Criscuolo and Sueur called this *evolutionary ethics*. Simple acts such as reducing meat consumption (Bègue and Treich, 2019; Espinosa et al., 2020) would have hugely positive impacts on animal ethics (reduction of animal suffering and use), environmental ethics (reduction of climate change and increase in biodiversity) and human health (reduction of cancer and cardiovascular diseases as well as a decrease in the number of new pathogens).

The moral consideration of animals also concerns animal research, regardless of whether our goals are fundamental or applied. A great amount of progress has been made regarding the 3Rs (Replace, Reduce, Refine) but researchers need to continue their efforts to reduce the number of animals they use and the suffering that animals endure. Specific guidelines exist now for research in the wild (Costello et al., 2016) and in the lab (Soulsbury et al., 2020). Surprisingly, animal behavior science remains on the sidelines, despite producing critical evidence on which many animal ethics arguments are based (Webb et al., 2019a). In this way, Patter and Blattner (2020) advance core principles to follow with animals: non-maleficence, beneficence and voluntary participation (Webb et al., 2019b). Economic or convenience euthanasia of animals should be not an option (Hayashi et al., 2013; Matsuzawa, 2016). Animals are not objects, and many species display forms of consciousness and sentience (Low et al., 2012). If we hope to change the habits of humans around the world, researchers must use their knowledge to be the pioneers whose behavior with non-human animals is identical to that they would show with persons possessing such consciousness. Researchers should not carry out research *on* animals, but rather *with* animals, and this mindset must be applied at the different levels of research (researcher, institution, reviewers, editors, funders; Field et al., 2019).

## Conclusions

A unanimous view that emerged several years ago is that we should no longer consider ourselves to be an element outside biodiversity, but rather a full component of it. We need to understand how we are interconnected to this world and its inhabitants. We must seek a new way of behaving toward domesticated species, find innovative means to replace our activities within ecosystems and live in symbiosis with other species (Criscuolo and Sueur). Instead of forcing animals to participate in certain work activities, we can use their remarkable socio-cognitive skills to interact with us and with other animal species (Bhattacharjee et al.). Indeed, ecological interactions between animal (and plant) species, whether it is symbiosis or competition, can be used for agriculture, farming or other services instead of using mechanical or chemical treatments that are harmful for human, animal or environmental health. However, animal behavior science is still insufficiently used. Evolutionary ethics thus proposes to cease differentiation between animal ethics and environmental ethics, and to replace human activities at the core of ecosystems. Non-human animals have always been important for human life due to the ecological, cultural and economic roles that they fulfill. However, one cannot work on animal welfare without involving human cultural aspects, making it difficult to impose universal standards of animal wellbeing, animal conservation and animal use (Kletty et al.; von Essen et al., 2020). Nevertheless, it is important to consider them as a part of our social and cultural capital rather than as material capital (Bourdieu, 1980; Throsby, 1999; Siisiainen, 2003). Parallels with previous human discriminations have been well documented (Kappeler, 1995; Dhont et al., 2020; Moffett, 2020) and non-human animals have to be considered as part of our societies (Donaldson and Kymlicka, 2011; Hoffman et al., 2018). This new animal consideration also involves us, the researchers. We should not continue to consider animals as simple inert objects for research but rather rethink our research and its scientific value in comparison with the number of animal lives we take (Costello et al., 2016; Webb et al., 2019a; Patter and Blattner, 2020; Soulsbury et al., 2020). Importantly, whatever the role of animals in our societies (pets, research, farm, etc.), their abuse is linked with psychopathological factors (Bègue, 2020), and respecting animals could lead us to see ourselves in a better light.

## Author Contributions

CS wrote a first draft. All authors contributed to the revision of the manuscript.

## Conflict of Interest

The authors declare that the research was conducted in the absence of any commercial or financial relationships that could be construed as a potential conflict of interest.
